# Clinical characteristics and prognosis of histological subtypes of papillary thyroid carcinoma in pediatric patients

**DOI:** 10.1530/EO-24-0078

**Published:** 2025-04-14

**Authors:** Junko Akaishi, Kiminori Sugino, Tetsuo Kondo, Wataru Kitagawa, Kenichi Matsuzu, Akifumi Suzuki, Chisato Tomoda, Ritsuko Okamura, Kiyomi Y Hames, Chie Masaki, Yoshiyuki Saito, Kana Yoshioka, Kosuke Inoue, Ryohei Katoh, Koichi Ito

**Affiliations:** ^1^ Department of Surgery, Ito Hospital, Tokyo, Japan; ^2^ Department of Human Pathology, University of Yamanashi, Yamanashi, Japan; ^3^ Department of Social Epidemiology, Graduate School of Medicine, Kyoto University, Kyoto, Japan; ^4^ Department of Pathology, Ito Hospital, Tokyo, Japan

**Keywords:** papillary thyroid carcinoma, pediatric thyroid carcinoma, histological subtypes, prognosis, surgery

## Abstract

The purpose of this study was to clarify the clinicopathological features and prognosis of histological subtypes of papillary thyroid carcinoma (PTC) in the pediatric population treated at a single institution. A total of 153 PTC patients ≤18 years of age who underwent initial surgery between 1979 and 2019 were investigated. There were 135 female and 18 male patients, with a mean age at the time of surgery of 16 (range, 8–18) years. The most common subtypes included classic PTC in 124 (81%), solid variant in 16 (10%), diffuse sclerosing variant in 7 (5%) and follicular variant in six (4%) according to the 5th edition of the WHO classification. At initial surgery, 49 patients (32%) had clinical lymph node metastases (cN1), 137 patients (90%) had pathological lymph node metastases (pN1), 73 patients (48%) had number of lymph node metastases (NLNMs) ≥10, 16 (10%) had gross extrathyroidal extension (ETE) and 18 (12%) had lung metastases. During a mean follow-up of 16 years, three (2%) patients died of their disease and 34 (25%) patients had recurrent disease. The 30-year cause-specific survival rate was 97.2%, and the 30-year disease-free survival (DFS) rate was 65.0%. On multivariate analysis, gross ETE, cN1 and NLNMs ≥10 identified as significant factors related to DFS (hazard ratio (HR) 4.13, confidence interval (CI) 1.48–9.96, *P* = 0.009; HR 2.34, CI 1.09–4.95, *P* = 0.0293; HR 2.81, CI 1.30–6.59, *P* = 0.008), but not histological subtype, were associated with disease recurrence. Histological subtypes were not associated with disease recurrence, but long-term follow-up of pediatric patients is necessary to investigate the biological characteristics.

**Synopsis:**

This retrospective study investigated the clinicopathological features and clinical outcomes of histological subtypes of PTC in a large series of pediatric patients treated at a single institution. It found that the prognosis of pediatric patients with PTC was excellent, but recurrence was common. In pediatric PTC, histological subtype did not affect survival and recurrence.

## Introduction

### Incidence and prognosis

The incidence of pediatric differentiated thyroid carcinoma (DTC) has been rising in recent years, with papillary thyroid carcinoma (PTC) accounting for most of the DTC cases (Vergamini *et al.* 2014, Qian *et al.* 2019, Lee *et al.* 2021). Compared with adult DTC, the biological behavior of pediatric DTC is different. The spectrum of genetic mutations of pediatric DTC is reported to differ from that of adult DTC (Cordioli *et al.* 2015). Pediatric PTC shows a higher prevalence of lymph node metastasis and distant metastasis, and a higher incidence of recurrence than adult PTC (Brink *et al.* 2000, Borson-Chazot *et al.* 2004, Collini *et al.* 2006, Markovina *et al.* 2014, Balachandar *et al.* 2016, Hay *et al.* 2018, Lee *et al.* 2021). However, pediatric PTC has an excellent long-term prognosis, with 30-year survival rates of 90–99% (Qian *et al.* 2019, Sugino *et al.* 2020). Several risk factors for recurrence gender (male), age, multifocality, gross extrathyroidal extension (ETE), preoperative lymph node metastasis and number of metastatic lymph nodes (NMLNs) have also been reported (Thompson & Hay 2004, Sugino *et al.* 2015, Jeon *et al.* 2018, Sugino *et al.* 2020). We have proposed that lobectomy may be sufficient as the initial surgical procedure for low-risk pediatric patients (Sugino *et al.* 2015, Sugino *et al.* 2020).

### Pathological diagnosis

According to the 5th edition of the WHO classification, there are many subtypes of PTC, with the diffuse sclerosing variant (DSV) and the solid variant of PTC common in the pediatric population (Juhlin *et al.* 2023). The cribriform-morular variant of PTC (CMV-PTC) was also common in the pediatric population, but the recent WHO classification defined CMV-PTC among the ‘thyroid tumors of uncertain histogenesis’ (Juhlin *et al.* 2023). Tall cell variant, columnar cell variant, DSV and the solid variant of PTC were classified as a ‘high-risk’ group than classical PTC, as in the adult criteria (Silver *et al.* 2011). It is uncertain whether the histological subtype of PTC affects the prognosis in the pediatric population. This study was conducted to clarify the clinicopathological features and clinical outcomes of histological subtypes of PTC in a large series of pediatric cases treated at a single institution in the past 40 years. To date, pediatric PTC has been treated using the same strategies as adult PTC. In 2015, the American Thyroid Association (ATA) published the first guidelines targeted for DTC children, those under 18 years of age, and classified pediatric patients into three categories (low-, intermediate- and high-risk groups), with risk stratification based on having persistent cervical disease and/or distant metastases after initial surgery (Francis *et al.* 2015). However, this guideline did not specify cutoff points for LN metastasis as minimal or extensive disease. The clinical utility of such risk stratification is limited. Therefore, the present study aimed to validate this risk stratification.

## Materials and methods

### Patients

A total of 21,355 PTC patients underwent initial surgery at Ito Hospital in Tokyo between 1979 and 2019. Of them, 153 (7%) PTC patients ≤18 years of age were evaluated. All PTCs were reviewed by experienced endocrine pathologists, TK and RK, co-authors of this study, blinded to patients’ outcomes, according to the 5th edition of the WHO classification (Juhlin *et al.* 2023). In the 4th WHO classification, CMV-PTC was classified as a subtype of PTC, but in the 5th WHO classification, it was included among ‘thyroid tumors of uncertain histogenesis’ (Juhlin *et al.* 2023). All patients were retrospectively staged according to the 8th TNM classification (Amin *et al.* 2017). All information used in the present study, including the patients’ characteristics, operative findings, postoperative treatment and follow-up, was collected from the patients’ medical records. Neck ultrasonography (US) and computed tomography (CT) have been routinely used preoperatively to evaluate neck disease and lung metastasis. Distant metastases were diagnosed by CT and whole body scintigraphy after total thyroidectomy. For all patients diagnosed with distant metastasis at initial surgery, radioactive iodine (RAI) therapy was performed within 6 months after initial surgery. All samples were obtained with informed patient consent and with approval from the Ito Hospital Institutional Review Board (approval no. 2018: 222). The protocol of this study was reviewed and approved by the institutional review board, and the study was performed in accordance with the Declaration of Helsinki.

### Follow-up

Postoperative follow-up examinations were usually performed at 1, 3, 6 and 12 months, and every 6 months thereafter. The serum thyroglobulin (Tg) level was routinely measured at every hospital visit. Whenever a gradual increase in the postoperative Tg level was observed in patients who had not undergone completion thyroidectomy, CT was performed. However, patients with positive Tg antibody were followed by CT and US, because the presence of Tg antibody interferes with the Tg immunometric assay and makes the Tg levels unreliable. If these examinations resulted in detection of metastasis, completion thyroidectomy was performed, followed by RAI scintigraphy. Postoperative thyrotropin (TSH) suppression therapy was performed selectively in patients at high risk, such as those with distant metastasis, gross extrathyroidal extension (ETE) and massive lymph node metastases (N1), but the patients’ TSH levels were not analyzed in this study according to the ATA guideline. The clinical response rate of distant metastasis to RAI therapy was evaluated according to the 2015 ATA guideline for adult DTC management (Haugen *et al.* 2016) and the Response Evaluation Criteria in Solid Tumors (RECIST) criteria, version 1.1 (Eisenhauer *et al.* 2009).

### Statistical analysis

Cause-specific survival (CSS), disease-free survival (DFS), lymph node recurrence-free survival (LNRFS) and distant metastasis-free survival (DMFS) rates were calculated using the Kaplan–Meier method. DFS rates were calculated in patients who underwent curative surgery without distant metastases at diagnosis. The impact of various factors on survival was analyzed by the log-rank test. Multivariate analyses of prognostic factors were based on the Cox proportional hazards model. A chi-squared test or Fisher’s exact test was performed for categorical variables. The Wilcoxon signed rank-sum test was performed for nonparametric continuous data. All *P*-values were two-sided, and values of *P* < 0.05 were considered significant. All statistical analyses were performed with computer software (JMP ver. 12.0; SAS Institute Inc., USA).

## Results

### Patients’ characteristic*s*

The patients’ characteristics are shown (Table 1). There were 135 female (88%) and 18 male patients (12%), with a mean age at the time of surgery of 16 (range, 8–18) years, and 5% (8 patients) were ≤10 years of age at diagnosis. There was no history of radiation exposure. At initial surgery, 49 patients (32%) had clinical lymph node metastases (cN1), 16 (11%) had gross ETE and 18 (12%) had distant metastases. Total thyroidectomy was performed in 55 (36%) patients and 98 (64%) patients underwent less than total thyroidectomy. Central lymph node dissection was performed in 42 patients (27%) and modified lateral lymph node dissection was performed in 109 patients (70%). A total of 137 (90%) patients had pathological lymph node metastases (pN1) and 73 patients (48%) had NLNMs ≥10. The median number of metastatic LNs was nine (0–58), and the cutoff for the number of metastatic LNs (NLNMs) by the ROC curve was ten (AUC 0.75) for predicting recurrence; the cutoff point showed sensitivity (0.31) and specificity (0.73).

**Table 1:** Clinical characteristics of the 153 pediatric patients with PTC.

Characteristic	*n* (%)	Range
Median age at diagnosis, y (range)		16 (8–18)
Sex		
Female	135 (88%)	
Male	18 (12%)	
Histology		
Classical	124 (81%)	
Solid	16 (10%)	
DSV	7 (5%)	
Follicular	6 (4%)	
Primary tumor size		
<40 mm	106 (69%)	24 (4–80) mm
>40 mm	47 (31%)	
Distant metastasis		
M0	18 (12%)	
M1	135 (88%)	
Clinical lymph node metastasis		
cN0	104 (68%)	
cN1	49 (32%)	
Pathological lymph node metastasis		
pN0 or pNX	16 (10%)	
pN1a	39 (25%)	
pN1b	98 (65%)	
Gross extrathyroidal extension		
Yes	16 (10%)	
No	137 (90%)	
Thyroidectomy		
Total (TTx)	82 (54%)	
Less than TTx	71 (46%)	
Lymph node dissection		
None	5 (3%)	
CND	41 (27%)	
MND	107 (70%)	
RAI therapy		
Ablation	21 (46%)	
100 mCi	25 (54%)	

DSV, diffuse sclerosing variant; Tx, thyroidectomy; CND, central node dissection; MND, modified neck dissection; RAI, radioactive iodine; PTC, papillary thyroid carcinoma.

### Histopathological characteristics

The histopathological characteristics are shown (Table 2). The most common subtypes included classic PTC in 124 (81%), solid variant in 16 (10%), DSV in 7 (5%) and follicular variant in six (4%). The subtypes of PTC accounted for only 29 (9%) patients of a small number of patients. The median primary tumor size was 24 (range, 4–80) mm.

**Table 2:** Comparison of patient characteristics.

		Classical *n* (%)	Variants *n* (%)	*P* value
Sex	Female	111 (90%)	24 (83%)	NS
	Male	13 (11%)	5 (17%)	
Age (years)	≤10 years	5 (4%)	3 (10%)	NS
	>10 years	119 (96%)	26 (90%)	
T (primary tumor size)	≤40 mm	104 (84%)	17 (59%)	NS
	>40 mm-diffuse*	20 (16%)	12 (41%)	
N (clinical lymph node metastasis)	cN0	88 (71%)	36 (74%)	NS
	cN1	36 (29%)	13 (26%)	
Massive extra-thyroidal invasion (ETE)	No	112 (90%)	25 (86%)	NS
	Yes	12 (10%)	4 (14%)	
Distant metastasis (M1)	No	110 (89%)	25 (86%)	NS
	Yes	14 (11%)	4 (14%)	
NMLNs	<10	66 (53%)	14 (48%)	NS
	≥10	58 (47%)	15 (52%)	
Multifocal	No	60 (48%)	12 (41%)	NS
	Yes	64 (52%)	17 (59%)	
Extent of thyroidectomy	Total (TTx)	78 (63%)	14 (47%)	NS
	Less than TTx	46 (37%)	15 (53%)	
Lymph node dissection	None/PCND	39 (31%)	7 (24%)	NS
	PMND	85 (69%)	22 (76%)	
RAI therapy	No	90 (73%)	17 (59%)	NS
	Yes	34 (27%)	12 (41%)	

* Diffuse, diffuse sclerosing variant.

ETE, extrathyroidal extension; pN, pathological lymph node metastasis; PTC, papillary thyroid carcinoma.

### RAI therapy and tyrosine kinase inhibitor (TKI) therapy

RAI therapy was performed in 46 of 82 (56%) patients who underwent total thyroidectomy containing completion to total thyroidectomy. All 18 patients with distant metastasis at initial surgery underwent RAI therapy. Of the ten patients who developed distant metastasis during follow-up, eight were treated with RAI therapy. Their disease in the lung was RAI-avid, and they underwent RAI therapy several times. The median total radiation dose was 100 (range, 30–700) mCi. The response rate for the 23 patients treated with RAI at 100 mCi or more was 57%. The remaining two with micronodular disease refused RAI therapy. No patients received TKI therapy.

### Clinical outcomes

**Figure 1:**
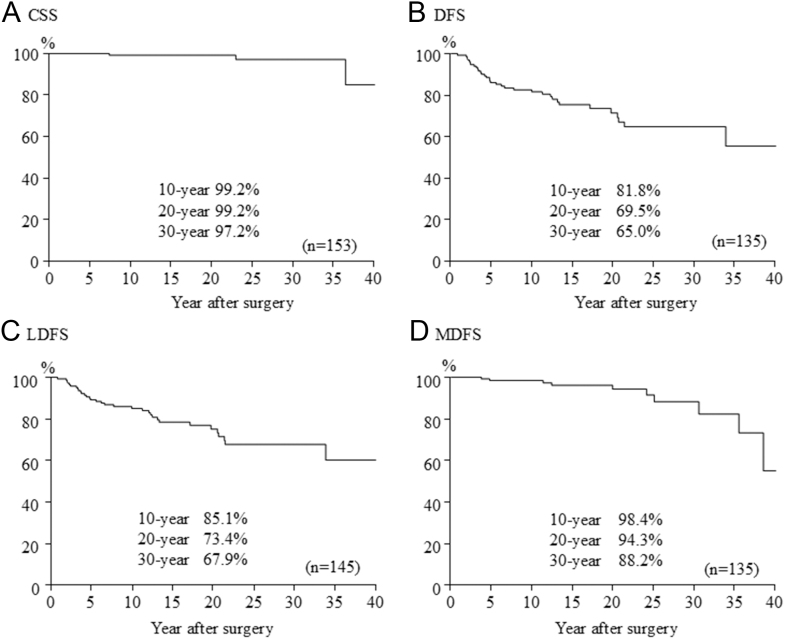
CSS, DFS, LNRFS and DMFS rates in all subjects.

**Figure 2:**
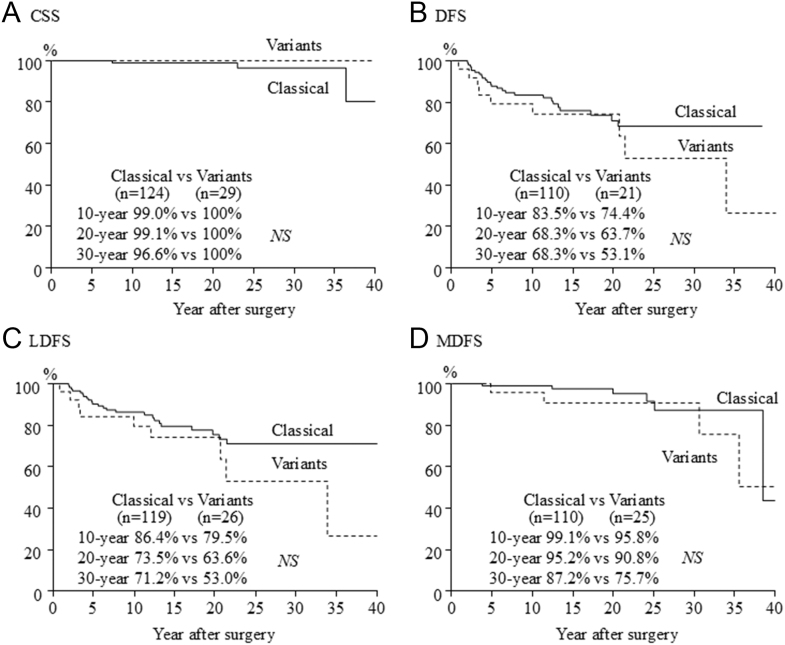
Comparison of CSS, DFS, DMFS and LNRFS rates between the classical PTC group and the variant group.

During a mean follow-up of 16 years, lung metastases were the cause of death of all three patients who died (Table 3). Recurrences were diagnosed in 37 patients. The recurrence was in lymph node metastases in 30 patients, remnant thyroid in four patients and both in one patient. Distant metastasis was detected at presentation in 18 patients and during follow-up in the other ten patients. Cumulative CSS, DFS, LNRFS and DMFS are shown (Fig. 1). The 10-, 20- and 30-year CSS rates were 99.2, 99.2 and 97.2%, respectively. The 10-, 20- and 30-year DFS rates were 81.8%, 69.5 and 65.0%, respectively. The 10-, 20- and 30-year LNRFS rates were 85.1, 73.4 and 67.9%, respectively, and the 10-, 20- and 30-year DMFS rates were 98.4, 94.3 and 88.2%, respectively. Furthermore, survival was compared between the classical PTC group and the variant PTC group (Fig. 2). The three fatal cases were all classical PTC cases. There were no significant differences between classical PTC and variants of PTC. The 30-year CSS rate was 96.6% in classical PTC and 100% in variant PTC. The 30-year DFS rates were 68.3% in classical PTC, 62.5% in solid variant, 57.1% in DSV and 57.1% in follicular variant. There were no significant differences between classical PTC and each variant of PTC.

**Table 3:** Clinical characteristics and treatment of the three fatal cases with lung metastasis.

No.	Age (y)	Sex	Histology	T	cN	pN	M	ETE	Dose of RAI therapy (mCi)	Follow-up (y)
1	9	M	Classical	T4a	cN1	pN1b	Lung	Trachea, RLN	100	22.9
2	12	F	Classical	T3	cN1	pN1b	Lung	None	100	36.4
3	17	F	Classical	T4a	cN0	pNx	Lung	Trachea, RLN	500	7.4

RLN, recurrent laryngeal nerve.

### Risk factor analysis and risk stratification

**Figure 3:**
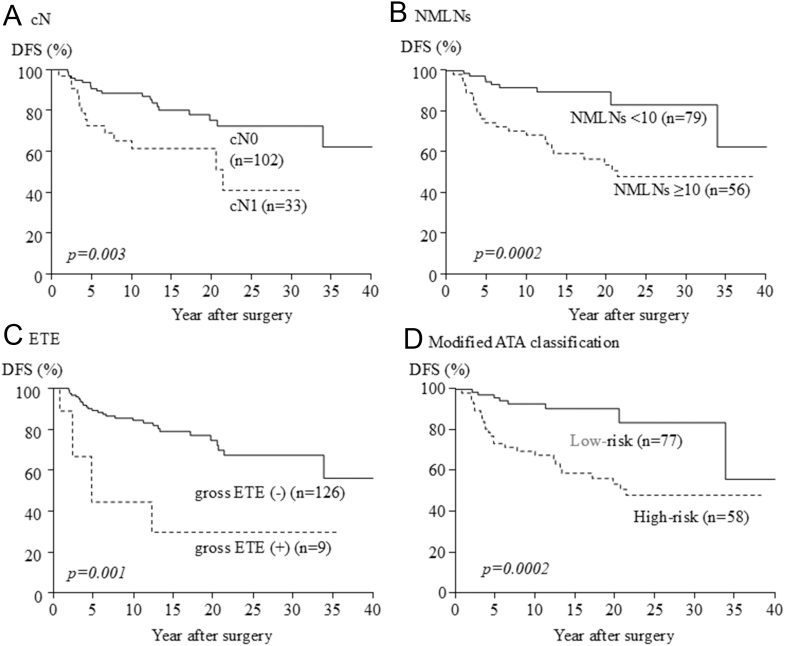
Comparison of DFS rates between patients with low-risk or high-risk group by modified ATA guideline.

Results of univariate and multivariate analyses to identify prognostic factors of recurrence are given in Table 4. On univariate analysis, gross ETE (*P* = 0.001), cN1 (*P* = 0.003) and NLNMs ≥10 (*P* = 0.0002) were found to be significant (Fig. 3A, B, C). On multivariate analysis, gross ETE, cN1 and NLNMs ≥10 were identified as significant factors related to DFS (hazard ratio (HR) 4.13, confidence interval (CI) 1.48–9.96, *P* = 0.009; HR 2.34, CI 1.09–4.95, *P* = 0.0293; HR 2.81, CI 1.30–6.59, *P* = 0.008). Based on this result, the patients were classified into two groups according to the presence of ETE, the status of cervical LN metastases and/or distant metastasis. The definitions were modified from the recent ATA guideline for DTC children shown in (Table 5). Patients with N0, NX or N1a disease or patients with minimal N1b with NLNMs <10 were assigned to the low-risk group. Patients with extensive N1b with NLNMs ≥10 or patients with gross ETE or patients with distant metastasis were assigned to the high-risk group. According to this original classification, 77 (50%) and 76 (50%) patients were assigned to the low- and high-risk groups, respectively. Of the 135 patients who underwent curative surgery without distant metastases at diagnosis, recurrence was observed in eight (10%) of the low-risk group and 26 (45%) of the high-risk group. The 10-year DFS rates were 92.9 and 67.9% in the low- and high-risk groups, respectively. The high-risk group had significantly higher disease recurrence than the low-risk group (*P* = 0.0002) (Fig. 3D).

**Table 4:** Univariate and multivariate analyses to identify prognostic factors of recurrence related to clinicopathological characteristics.

Factors related to DFS	Univariate			Multivariate		
	HR	CI	*p*	HR	CI	*p*
Gross extrathyroidal extension (ETE)	3.97	1.48–9.01	0.001	4.13	1.48–9.96	0.009
Clinical lymph node metastasis (cN1)	2.73	1.34–5.43	0.003	2.34	1.09–4.95	0.0293
NLNMs ≥10	3.82	1.84–8.69	0.0002	2.81	1.30–6.59	0.008

NLNMs, number of lymph node metastases; DFS, disease-free survival; HR, hazard ratio; CI, confidence interval.

**Table 5:** Definitions of the modified recent ATA guideline for DTC children.

Category	Definition	Total	Incidence of death	Incidence of recurrence
		*n* (%)	*n* (%)	*n* (%)
Low	N0 or NX or N1a or minimal N1b with NLMs<10	77 (50%)	0 (0%)	8/77 (10%)
High	Extensive N1b with NLNMs ≥10 or gross ETE or distant metastasis	76 (50%)	3 (4%)	26/58 (45%)

DTC, differentiated thyroid carcinoma.

## Discussion

The ATA guidelines defined children as those under 18 years of age and proposed treatment strategies for DTC in children. In our previous study, we suggested that an age cutoff of <15 years rather than <19 years may be more suitable in pediatric DTC (Francis *et al.* 2015). Therefore, in the present study, PTC patients under 18 years of age were defined as children. The study in the United States from 1973 to 2013 reported that, of 1,806 patients with thyroid cancer who were younger than 20 years, 1,454 (80.5%) were female, the male-to-female ratio was 1:4 and most patients were aged 15–19 years (Qian *et al.* 2019). In the present study, the male-to-female ratio was 1:8, and there were more female patients. Distribution by age showed a trend toward an increase in the late teens. Lymph node metastasis and distant metastasis were frequent at the time of diagnosis.

In the present study, 29 (19%) patients had a subtype, solid variant in 16 (10%) and DSV in seven (5%). However, both variants had an excellent prognosis, similar to that of classical PTC in pediatric PTC patients, possibly due to the positive effect of younger patient age. The solid variant is more common in patients with a history of exposure to ionizing radiation (Nikiforov & Gnepp 1994). Vuong *et al.* reported a meta-analysis of the solid variant of PTC, and vascular invasion, extraglandular invasion and distant metastasis were more common, and recurrence and mortality rates were higher than that in the other types of PTC (Vuong *et al.* 2018). Ohashi *et al.* reported that DFS was shorter than that of classical PTC (Ohashi 2020). In the present study, all 16 patients with solid variant of PTC did not have a history of exposure to neck radiation. All patients were alive, and five patients had recurrence. The 30-year CSS and DFS rates were 100 and 62.5%, respectively. The prognosis for the solid variant was the same as for classical PTC.

In 1985, Vickery *et al.* described the DSV of PTC (Vickery *et al.* 1985). This variant is rare, accounting for 1–2% of PTC cases, and it is more common in young women in their teens and 20s (Vickery *et al.* 1985, Fujimoto *et al.* 1990, Koo *et al.* 2009). More extensive lymph node metastasis, extrathyroidal invasion and distant metastasis are seen than in classical PTC (Fukushima *et al.* 2009, Pillai *et al.* 2015, Vuong *et al.* 2017). Outcomes for DSV were good, but recurrence was more common than for classical PTC (Akaishi *et al.* 2015). Balachander *et al.* reported that event-free survival was not associated with histological subtype (Balachandar *et al.* 2016). In the present study, the three fatal cases all had classical PTC, and histological subtype did not affect survival and recurrence. The subtypes of PTC accounted for only 29 (9%) patients of a small number of patients, which may not have been reflected in a difference in prognosis.

Recent ATA guidelines for DTC children did not specify cutoff points of the number of LN metastases and the clinical utility of such risk stratification is limited (Francis *et al.* 2015). In the present study, risk factors related to DFS were gross ETE, clinical lymph node metastasis (cN1) and NLNMs ≥10 on multivariate analysis. Therefore, we have modified the ATA guideline to include the presence of ETE and the status of cervical LN metastases and/or distant metastasis, and divided the patients into two categories, reflecting the results of the multivariate analysis. The DFS rate of the high-risk group was significantly lower than that of the low-risk groups (*P* = 0.0002).

Even with higher prevalence of lymph node metastasis and distant metastasis, the long-term prognosis is excellent in pediatric patients with PTC. This is considered due to the better response to RAI therapy in pediatric PTC patients with lung metastasis than in adults (Pawelczak *et al.* 2010). However, secondary primary malignancy has been reported to be high in pediatric DTC patients treated with RAI therapy (Marti *et al.* 2015, Zhao *et al.* 2021, Pasqual *et al.* 2022). However, large amounts of data from other centers have not shown an increase in breast cancer or a secondary cancer risk with RAI therapy (Kim *et al.* 2022, Nappi *et al.* 2022).

Most genetic abnormalities in PTCs are concentrated in the MAPK pathway, with *RET* gene rearrangements, *BRAF* point mutations (*BRAF*-V600E) and *NTRK* gene rearrangements (Kondo *et al.* 2006). *RET* gene rearrangements are the most frequent in PTCs in children and young adults, and *BRAF* mutations are less frequent than in adults (Kebebew *et al.* 2007). *RET/PTC3* was reported frequently in pediatric thyroid cancers after the Chernobyl accident, suggesting an association with childhood thyroid cancer after radiation exposure of different types of *RET/PTC* (Nikiforov *et al.* 1997). DSV has a higher frequency of *RET/PTC* rearrangement, but *BRAF* mutation is rare (Nakazawa *et al.* 2005). In 52/93 (55.9%) pediatric PTC patients, a fusion gene, 20 different types of *RET*, *NTRK3*, *ALK*, *NTRK1*, *BRAF and MET* fusions were detected, and *RET* fusions were associated with more frequent lymph node and distant metastases, and *NTRK3* fusions were associated with the follicular variants of PTC (Pekova *et al.* 2020). In Japan, molecular-targeted agents have been indicated for advanced thyroid carcinoma that is either unresectable or resistant to RAI therapy since 2014. Subsequently, BRAF/MEK inhibitors, RET inhibitors and TRK inhibitors were approved for advanced thyroid carcinoma. In thyroid carcinomas, genetic testing is recommended to determine the indication for therapeutic agents tied to specific genetic abnormalities. In the present study, the three fatal cases due to pulmonary metastases could not undergo genetic testing and could not be treated by TKI therapy because TKI had not yet been approved at that time.

Several limitations must be considered in interpreting the results of this retrospective study. Our hospital is a single center without a pediatric department, which may introduce bias in the patient population. Further study is needed to evaluate more cases. The indications for RAI therapy in Japan are limited to patients with gross ETE, massive lymph node metastasis or distant metastasis.

## Conclusions

The prognosis of pediatric patients with PTC was excellent, but recurrence was common. In pediatric PTC, histological subtype did not affect survival and recurrence. Long-term follow-up of pediatric patients is necessary to investigate the biological characteristics. In particular, it is important to understand the special characteristics of histological subtypes.

## Declaration of interest

The authors declare that there is no conflict of interest that could be perceived as prejudicing the impartiality of the work reported.

## Funding

This work did not receive any specific grant from any funding agency in the public, commercial or not-for-profit sector.

## Author contribution statement

JA designed the study, carried out data analysis and wrote the original draft. KS, TK and RK designed the study. WK, KM, AS, CT, RO, KH, CM, YS, KY, KI and KI reviewed and edited the article. All authors revised and approved the final version of the article.
